# Screening of β -damascenone-producing strains in light-flavor *Baijiu* and its production optimization *via* response surface methodology

**DOI:** 10.3389/fmicb.2022.1067671

**Published:** 2022-11-29

**Authors:** Jie Tang, Bin Lin, Wei Jiang, Qun Li, Liping Zhu, Gang Zhang, Qianjin Chen, Qiang Yang, Shengzhi Yang, Shenxi Chen

**Affiliations:** Hubei Key Laboratory of Quality and Safety of Traditional Chinese Medicine and Health Food, Jing Brand Co., Ltd., Daye, China

**Keywords:** *Baijiu*, β-damascenone, statistical optimization, *Wickerhamomyces anomalus*, Box-Behnken design, Plackett-Burman design

## Abstract

As a C13-norisoprenoid aroma substance, β-damascenone is a highly important aromatic compound and an active constituent. The purpose of this study was to investigate the change law of β-damascenone during the light-flavor *Baijiu* brewing process, and screen the indigenous microbial strains that produce this compound and optimize fermentation parameters for improving β-damascenone production using a statistical approach. In this project, *Wickerhamomyces anomalus* YWB-1 exhibited the highest producing activity of β-damascenone. Fermentation conditions were optimized for β-damascenone production using a one-factor-at-a-time (OFAT) approach. A Plackett-Burman design was subsequently adopted to assess the effects of initial pH, incubation temperature, inoculum size, fermentation period, and original Brix degree. Analysis of variance (ANOVA) showed that the correlation coefficient (*R*^2^) of the executive model was 0.9795, and this value was significant (*p* < 0.05). Three significant variables were optimized at three different coded levels using a Box-Behnken design (BBD) of response surface methodology (RSM). Here, 7.25 μg/L β-damascenone was obtained under the following optimum conditions: initial pH of 3.31, original Brix degree of 10.53%, and fermentation period of 52.13 h. The yield was increased 3.02-fold compared with that obtained under unoptimized conditions. This information is conducive to the control of flavor production by regulating variable parameters in *Baijiu* fermentation.

## Introduction

As a C13-norisoprenoid aroma substance, β-damascenone is one of the most important natural aromatic compounds. Although β-damascenone is the essential aroma component of tea, grapes, roses, and tobacco, it is also frequently encountered as a vital flavor contributor in alcoholic beverages (Rodríguez-Bustamante and Sánchez, 2007; Sefton et al., 2011). Due to its low threshold (0.002 μg/L in water) and high odor activity value (OAV), β-damascenone has already been described having as baked/cooked apples, honey, and flowery fruity aromas in previous research (Mendes-Pinto, 2009; Tomasino and Bolman, 2021). Furthermore, β-damascenone also exhibits interesting biological activities, including inhibition of the expression of proinflammatory cytokines and leukocyte adhesion molecules and the prevention of sunburn and skin cancer (Uddin et al., 2012; Pan et al., 2019).

With over thousands of years of history, light-flavor *Baijiu* (also referred to as Chinese light-flavor liquor) is one of three major flavor types (sauce-, strong-, and light-flavor) of *Baijiu*. *Baijiu* is processed through some basic stages, including raw material handling, grain steeping, grain steaming, grain cooling, starter addition, grain blending, fermentation, and distillation (Wang et al., 2020). Light-flavor *Baijiu* is mainly generated by complex microbial communities *via* a spontaneous fermentation process (Lin et al., 2022). Interestingly, these microbes produce many flavor compounds, including alcohols, aldehydes, acids, esters, terpenes, and ketones, which are critically related to the quality and value of *Baijiu*, although they only represent 1 to 2% (v/v) of whole *Baijiu* (Jin et al., 2017). β-damascenone, as a flavor compound, makes important contributions to the aroma of *Baijiu* (Wang J. et al., 2021; Wang Z. et al., 2021). β-damascenone is a breakdown product of different aglycone and neoxanthin formed by enzymatic and acid-catalyzed hydrolysis of certain hydroxylated precursors and their glycoconjugates in grapes and their respective wines (Daniel et al., 2008; Tomasino and Bolman, 2021). Supplementary Figure 1 showed the related content of the generation pathway of β-damascenone (Sun T. H. et al., 2022). In addition, β-damascenone can also be produced by some non-*Saccharomyces* yeasts during the fermentation processes of wine and beer, such as *Cyberlindnera saturnus*, *Debaryomyces hansenii*, *Hanseniaspora uvarum*, *Metschnikowia pulcherrima*, and *Wickerhamomyces anomalus* (Ugliano et al., 2006; Swangkeaw et al., 2010; Lloyd et al., 2011; Methner et al., 2022). However, to date, β-damascenone in *Baijiu* has rarely been reported. Wu et al. (2015) investigated the effects of various indigenous yeast species on the formation of terpenoids in Chinese light-flavor *Baijiu* as well as the yeasts that could produce β-damascenone, including *Saccharomyces cerevisiae*, *W. anomalus*, *Pichia kudriavzevii*, *Hanseniaspora osmophila*, *Pichia fermentans*, *Pichia membranifaciens*, and *Trichosporon asahii*. However, the fermentation conditions under which the yeast strains produced this substance have not been explored.

It is well known that the medium formulation and fermentation conditions play an important role in the production of microbial metabolites, affecting the quality and yield of the product. The production of β-damascenone is largely affected by environmental abiotic factors, such as nutrient composition, pH, and fermentation temperature. Therefore, selecting suitable variables and identifying the optimum value of each variable are crucial for β-damascenone production. A one-factor-at-a-time (OFAT) approach is commonly performed to optimize the medium variables to enhance microbial metabolites, and this method can effectively reduce the number of variables for subsequent factorial design (Singh et al., 2011). In addition, OFAT provides additional benefits by arranging the expected size of the experimental factors and main effects into the factorial design (Myers et al., 2009). However, OFAT is not guaranteed to achieve optimal conditions given its low accuracy, which is determined by the large number of experimental runs and the inability to determine the interactions between variables. Therefore, response surface methodology (RSM) is widely used to select important factors and obtain a level of optimization coupled with Plackett-Burman (PB) design (Wang et al., 2011). RSM is an effective statistical tool that is used to maximize a system’s performance by optimizing the significant variables. Moreover, the RSM can determine the main effects of variables and their interaction on the response, obtain optimum operating conditions from limited experimental results, and predict the response value for variables. Thus, the combination of these methods can be used in *Baijiu* fermentation to optimize the variable conditions for producing β-damascenone to improve the content of β-damascenone in *Baijiu*.

In this study, β-damascenone-producing strains in *Baijiu* were screened for the first time, and fermentation parameters for β-damascenone were optimized through a submerged fermentation process in cereal extract medium. PB was applied as a fractional factorial design to assess the significance of variables in the microbial fermentation process. Significant variables were further evaluated to assess their role in increasing the production of β-damascenone based on the quadratic polynomial model using the Box-Behnken Design (BBD) of RSM. This study clarifies the species of β-damascenone-producing strains of *Baijiu* and provides microbial sources for improving key aroma and health components in light-flavor *Baijiu*.

## Materials and methods

### Reagents and samples

β-carotene, β-damascenone, and L-menthol in high purity (≥97%) were purchased from Sigma Aldrich Co., Ltd. (Shanghai, China). Anhydrous sodium sulfate (Na_2_SO_4_) and sodium chloride (NaCl) were procured from Sinopharm Chemical Reagent Co., Ltd. (Shanghai, China). Dichloromethane (CH_2_Cl_2_) and ethanol were purchased from Thermo Fisher Scientific Co., Ltd. (Beijing, China). Ultrapure water was obtained from Purelab Ultra (Veolia, France). All chemicals were of analytical purity or higher.

β-carotene solution (50 mg/L) was produced as follows: β-carotene standard (5 mg) was dissolved in 10 mL dichloromethane, and then Tween-80 (1 g) was added to emulsify after complete dissolution under dark conditions. After the dichloromethane was completely evaporated, sterile distilled water (100 mL) was added, mixed evenly, and placed in a 100-mL brown volumetric flask.

Sampling was collected in a light-flavor *Baijiu* factory (Hubei, China) in 2021. The production process of light-flavor *Baijiu* has been described in detail in our previous research and the fermentation period was 14 days (Tang et al., 2022). The selected samples included *Jiuqu*, saccharified grains, and grains fermented for 0, 1, 2, 3, 4, 5, 7, 9, 11, and 14 days. These samples were termed J0, S1, D0, D1, D2, D3, D4, D5, D7, D9, D11, and D14, respectively. Each sample had three biological replicates. A total of 36 samples were obtained. These samples were stored at 4°C until further analysis.

### Isolation and identification of yeasts

Samples (10 g) were blended with 90 mL sterile saline (0.85% NaCl) and soaked at 4°C for 30 min. Portions (200 μL) of each of the resulting suspensions were spread on β-carotene-containing growth agar plates, which were prepared by adding β-carotene solution (50 mg/L) obtained above at a volume ratio of 20% into standard nutrient medium [30 g/L sucrose; 0.03 g/L yeast extract; 1 g/L KH_2_PO_4_; 0.5 g/L MgSO_4_; 3 g/L NaNO_3_; 0.01 g/L Fe_2_SO_4_; 0.5 g/L KCl; 6.7 g/L yeast nitrogen base (YNB) medium; 20 g/L agar] (Zhao et al., 2018). The color of the agar plates was assessed daily, and β-carotene-degrading strains were visually detected by color fading after 3–5 days.

The strains obtained above were isolated and purified and then inoculated into a colored liquid medium containing β-carotene [β-carotene solution (20%, v/v); 30 g/L sucrose; 0.03 g/L yeast extract; 1 g/L KH_2_PO_4_; 0.5 g/L MgSO_4_; 3 g/L NaNO_3_; 0.01 g/L Fe_2_SO_4_; 0.5 g/L KCl, 6.7 g/L YNB medium] for cultivation. Strains in the faded yellow liquid medium were selected after 5 days.

For identification, the fungal Internal Transcribed Spacer (ITS) rDNA region was amplified, sequenced, and aligned to publicly available sequences in the National Center for Biotechnology Information (NCBI) database using the Basic Local Alignment Search Tool (BLAST). The whole DNA genome of the yeast isolates was extracted using an extraction kit (DNA Extraction Kit, Tiangen Scientific, Beijing, China). ITS1 (5′-TCCGTAGGTGAACCTGCGG-3′) and ITS4 (5′-TCCTCC GCTTATTGATATGC-3′) primers were used to amplify the ITS rDNA region gene (Allahkarami et al., 2021). Polymerase chain reaction (PCR) was performed using the following thermal cycling conditions: predenaturation at 94°C for 5 min; 35 cycles of denaturation at 94°C for 30 s, annealing at 52°C for 50 s, and extension at 72°C for 60 s; and a final extension at 72°C for 10 min.

### Fermentation conditions

The fermentation medium was prepared from ground cereal (sorghum), which was the main raw material for brewing light-flavor *Baijiu* brewing. Two kilograms of sorghum flour was combined with 8 L of deionized water and steamed at 100°C for 30 min. The mixture was then saccharified at 60°C for 24 h followed by the addition of glucoamylase. The resulting mixture was centrifuged and filtered to obtain the supernatant. The specific sugar content and pH of the medium were adjusted according to the test situation.

A loopful of yeast cultures was inoculated into liquid yeast peptone dextrose (YPD) medium at 30°C for 24 h. These yeast seed cultures (2% v/v) were inoculated in 250-mL Erlenmeyer flasks with 50 mL of sterile fermentation medium (sorghum extract medium with 5.0 of initial pH and 8.0% of original Brix). Fermentation was conducted at 30°C for 96 h in the stationary state. Supernatants of the fermentation broth were obtained to determine the β-damascenone content. Each experiment was performed in triplicate.

### Optimization of fermentation conditions using the one-factor-at-a-time approach

The OFAT approach was used to determine the culture conditions of β-damascenone production, such as inoculum size, initial pH, original Brix, incubation temperature, medium volume, and incubation period. The experimental factors and their associated levels for medium conditions using OFAT experiments were shown in Supplementary Table 1 in the Supplementary material. Each experiment was performed in triplicate, and the mean value calculated was taken as the β-damascenone content.

### Screening for significant variables using a Plackett-Burman design

A PB design was used to determine important fermentation conditions for β-damascenone production based on the OFAT analysis results. Taking the β-damascenone content of the fermentation broth supernatant as the response value, a two-level factorial design with five factors and two levels of 1/2 was used. In this experimental design (Supplementary Table 2), five fermentation conditions (A: initial pH; B: incubation temperature; C: inoculum size; D: incubation period; and E: original biomass) were screened at two levels [low (−) and high (+)].

Analysis of variance (ANOVA) was performed to generate regression coefficients, prediction equations, and case statistics. The *F*-value was applied to examine the significance of each factor. The β-damascenone content was measured in triplicate, and the mean values were shown as response R. The steepest climb experimental design was used to further determine the optimal parameters of the significant factors affecting the production of β-damascenone by determining the optimal response value area and establishing a response surface fitting equation.

### Optimization of significant factors using a Box-Behnken design

To investigate the effects of different fermentation parameters on the β-damascenone content of fermentation broth supernatant fermented with the yeast strain, the BBD was applied. A face-centered central composite design with three independent factors and five replications of the center point was chosen based on PB and the steepest climb experimental design. As shown in Supplementary Table 3, the three variable factors were initial pH (2.5–4.5), original Brix (5–15%), and fermentation period (16–80 h). Therefore, a total of 17 fermentation trials were performed. This design resulted in a center point with the factors of fermentation time at 48 h, initial pH of 3.5, and original Brix of 10.0%. Variable optimization was achieved based on the regression equation, and the optimized values were observed on a three-dimensional response surface plot.

The statistical significance of the model was verified by applying ANOVA, and the lack of fit was taken into account to estimate the model. In general, the model significance was judged using Fisher’s *F*-test and its associated probability (F), and the polynomial model was determined by the coefficient of assurance (*R*^2^) and balanced *R*^2^ (Bhaturiwala et al., 2022). Further three-dimensional response surface plots and corresponding contour maps were used to illustrate the relationship between the responses and the experimental levels of significant variables.

### Quantitative analysis of β-damascenone using liquid-liquid microextraction combined with gas chromatography-mass spectrometry

β-damascenone was extracted using CH_2_Cl_2_ as the organic solvent, and L-menthol (100 mg/L) was used as the internal standard. Solid samples (10 g) were added to 25 mL sterile saline (0.85% NaCl and 1% CaCl_2_), ultrasonically treated for 30 min in an ice-water mixture, and then centrifuged at 8,000 × *g* for 5 min (4°C) to obtain supernatants. For liquid samples, yeast fermentation broths were centrifuged at 8,000 × *g* for 5 min (4°C). For the quantitation of β-damascenone, CH_2_Cl_2_ (2 mL), internal standard (20 μL), and NaCl (7 g) were added to the supernatants (20 mL). The mixture was stirred for 10 min at ambient temperature and sonicated in an ice bath for 10 min. The above obtained sample was centrifuged and refrigerated overnight to separate the organic phase. Next, the lower organic phase was collected and dried over anhydrous sodium sulfate. The extracted organic phase was analyzed using a gas chromatography-mass spectrometry (GC-MS) system (Agilent Technologies Inc., Santa Clara, CA, USA) equipped with a DB-FFAP column (30.0 m × 0.25 mm × 0.25 μm, J&W Scientific, Santa Clara, CA, USA). The standard solution was made from aqueous alcohol solution and applied to construct a standard curve. All standard solutions were analyzed in the same manner as described for samples. The conditions for GC-MS were based on a previously reported method (Sun X. Z. et al., 2022).

### Statistical analysis

The experimental design and subsequent statistical analysis of the obtained results were obtained by using a trial version of Design-Expert 8.0 (Stat-Ease; PA, USA). ANOVA was used to test for significant differences in β-damascenone content, which was further assessed using the Duncan test (*p* < 0.05) with Statistical Product Service Solutions (SPSS) 19.0 software (SPSS, Chicago, IL, USA). The remaining graphs were generated by Origin 2018 (OriginLab Corporation, Northampton, MA, USA).

## Results and discussion

### Change in β-damascenone in the light-flavor *Baijiu* fermentation process

**FIGURE 1:**
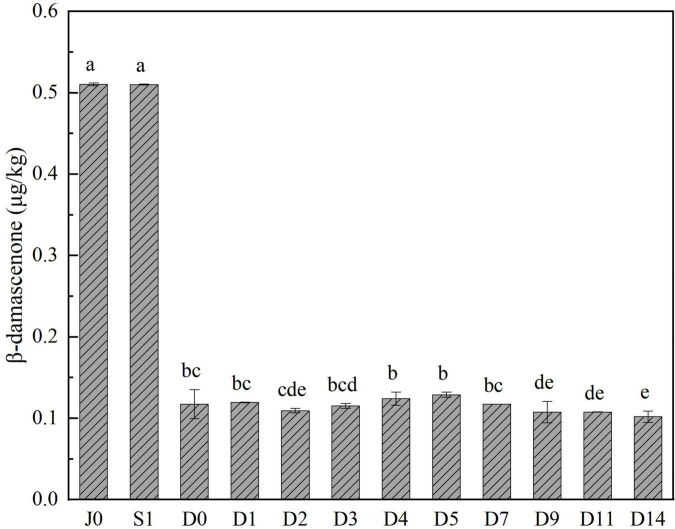
Change of β-damascenone contents during light-flavor *Baijiu* brewing process. Values (means ± SD, *n* = 3) with different letters indicate different significance at *p* < 0.05 (Duncan’s test).

To clarify the source of this substance and facilitate strain screening, the changes in β-damascenone in *Jiuqu*, saccharified grains, and fermented grains from an industrial scale light-flavor *Baijiu* fermentation were analyzed. As shown in Figure 1, the β-damascenone concentrations in all samples were very low, not exceeding 1 μg/kg. However, the concentrations of β-damascenone in *Jiuqu* and saccharified grains were higher than those in fermented grains, indicating that the fermentation process did not further promote the production of β-damascenone. It is possible that β-damascenone in light-flavor *Baijiu* originated from *Jiuqu* and saccharified grains, which provide the ideal sample source for screening strains with high β-damascenone yield. Thus, the separation and screening of β-damascenone-producing isolates from *Jiuqu* and saccharified grains were performed.

### Isolation and identification of the yeasts present in *Jiuqu* and saccharified grains

Neoxanthin, as the precursor of β-damascenone, is difficult to obtain on the market, so one of its structural analogs, β-carotene, was added to the medium as a precursor to screen β-damascenone-producing strains (Zorn et al., 2003; Timmins et al., 2020). The primary screening of the target strains was performed using a two-step method. First, some strains were obtained using β-carotene-containing growth agar plates. Second, the above-obtained strains were inoculated into yellow liquid medium containing β-carotene to screen the initial target strains by observing the change in the color of the medium (Supplementary Figure 2). In total, 35 strains of yeasts were isolated and identified from *Jiuqu* and saccharified grains based on the above two-step separation method (Table 1). These original yeasts were further categorized into 12 species by blasting against the NCBI database, including *S. cerevisiae*, *W. anomalus*, *Saccharomycopsis fibuligera*, *P. kudriavzevii*, *Pichia manshurica*, *Hyphopichia burtonii*, *Clavispora lusitaniae*, *Candida apicola*, and *Meyerozyma guilliermondii*. *Pichia*, *W. anomalus*, and *S. cerevisiae* were identified as the predominant species of yeasts in light-flavor *Baijiu*, which could synthesize alcohols and esters that contributed to the flavor of light-flavor *Baijiu* (Tu et al., 2022). *Pichia*, *W. anomalus*, and *S. cerevisiae* and accounted for 28.57, 20, and 14.29% of the yeast population, respectively, in this study. These results indicated that many strains in *Jiuqu* have the potential to produce β-damascenone.

**TABLE 1:** Identification results of different types of yeast.

Strain no.	Species
YDMT-1, YDMT-2, YG-16, YWB-6, YT-1	*Saccharomyces cerevisiae*
YDMT-5, YDMT-6, YDMT-7, YWB-7	*Pichia kudriavzevii*
YDMT-8, YDMT-9, YDMT-10, YDMT-11, YWB-14, YWB-2, YWB-3	*Wickerhamomyces anomalus*
YDMT-3, YWB-4	*Saccharomycopsis fibuligera*
YWB-8	*Pichia guilliermondii*
YWB-9, YDMT-12, YT-4	*Pichia manshurica*
YDMT-13	*Pichia fabianii*
YDMT-14	*Millerozyma farinosa (Pichia farinose)*
YDMT-15	*Hyphopichia* sp.
YDMT-16, YDMT-17, YDMT-18	*Hyphopichia burtonii*
YWB-10, YWB-11	*Clavispora lusitaniae*
YWB-12, YWB-13	*Candida apicola*
YDMT-19, YT-2, YWB-5	*Trichosporon asahii*

### Evaluation of indigenous yeast strains in sorghum extract medium

**FIGURE 2:**
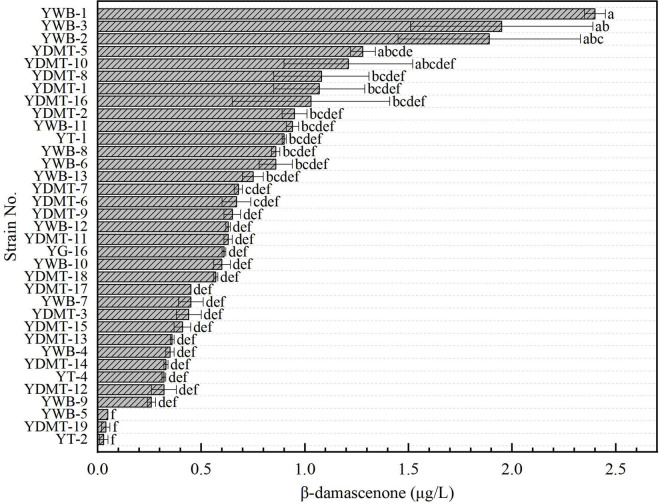
The β-damascenone concentrations of yeast supernatants. Values (means ± SD, *n* = 3) marked with the different lowercase letters represent significant difference (*p* < 0.05, Duncan’s test).

To further assess the fermentation performance of the indigenous yeasts, 35 yeasts were subsequently tested to screen the strains with high yields of β-damascenone by fermentation in sorghum extract medium. As shown in Figure 2, the contents of β-damascenone produced by most yeast strains ranged from 0.6 to 2.4 μg/L. In general, the strains belonging to *W. anomalus* exhibited good performance with high levels of β-damascenone production. Among these strains, *W. anomalus* YWB-1 produced the highest β-damascenone content. *W. anomalus* exhibits increased β-glucosidase activity compared with other non-*Saccharomyces* yeasts, enhancing the production of β-damascenone during wine fermentation (Li et al., 2020; Huang et al., 2021). In addition, different strains of yeasts belonging to the same species exhibited differences in β-damascenone production. For example, YWB-1, YDMT-8, YDMT-9, YDMT-10, YWB-2, and YWB-3 yielded significant differences in β-damascenone content.

### Fermentation conditions optimization through one-factor-at-a-time

**FIGURE 3:**
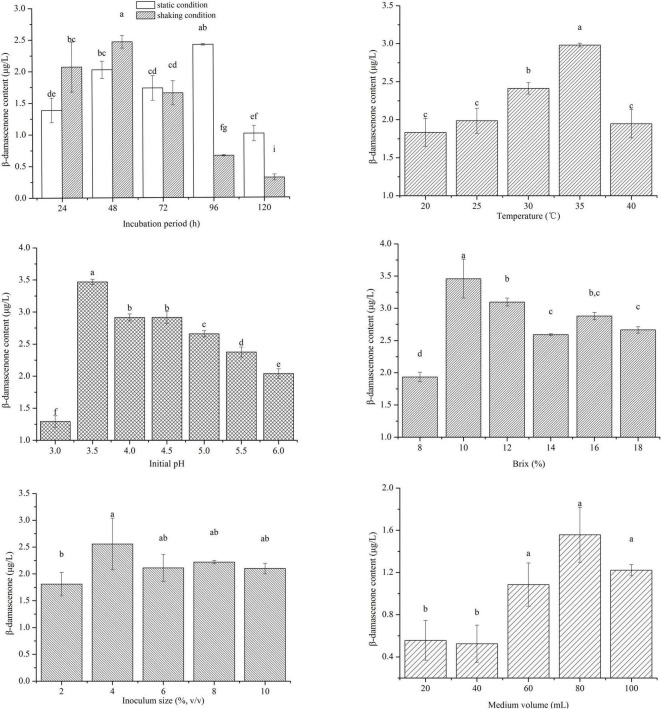
Effect of culture conditions on β-damascenone production by one-factor-at-a-time (OFAT). Values marked with the same lowercase letters represent no significant difference (*p* < 0.05, Duncan’s test).

The initial fermentation conditions of *W. anomalus* YWB-1 included static fermentation at 30°C in sorghum extract medium with an initial pH of 5.0 and an original Brix of 8.0% for 96 h. The best single factor was obtained by evaluating the variables using OFAT to select the optimal medium composition and fermentation conditions for the factorial design. Each selected variable was evaluated to determine its optimal value while holding all other experimental factors constant. The effects of different fermentation variables on β-damascenone production are shown in Figure 3.

The results showed that β-damascenone production reached the maximum value at 48 h under shaking conditions, whereas the maximum value was reached at 96 h under static conditions. This result indicated that shaking conditions accelerated the growth and metabolism of yeast, which resulted in shorter incubation times compared with those noted for static conditions. Fermentation time had a significant effect on β-damascenone production through microbial metabolism. However, when the fermentation time exceeded a certain number of hours, the production of β-damascenone did not increase, which was consistent with findings regarding other aroma compounds in Chinese *Baijiu* (Li et al., 2020; Tang et al., 2022).

The volatile composition was significantly affected by the temperature of fermentation in wine (Izquierdo-Cañas et al., 2020). The β-damascenone content first increased and then decreased with increasing culture temperature, and the highest values of this compound were obtained when the fermentation temperature was 35°C. Additionally, fermentation at a temperature that is too high will restrict yeast growth and reduce β-damascenone content (Methner et al., 2022). Thus, the proper temperature could improve β-damascenone production.

Previous research has shown that β-damascenone is formed by acid-catalyzed hydrolysis of certain hydroxylated precursors and their glycoconjugates (Lloyd et al., 2011), so the initial pH of the medium plays an important role in the formation of this compound. The highest content of β-damascenone was produced when the yeast was fermented at the initial pH of 3.5. Proper acidity could improve the formation of β-damascenone. However, a lower initial pH caused less yeast growth, and a higher initial pH also had adverse effects on β-damascenone production by decreasing acid-catalyzed glycoside hydrolysis (Gijs et al., 2002; Methner et al., 2022).

For non-alcoholic beer, the original gravity (similar to Brix) and pitching rate (similar to the inoculum size) have a demonstrable influence on the production of (E)-β-damascenone during fermentation (Methner et al., 2022). Here, the original Brix represents the content of available sugar in the cereal raw material extract medium and plays a crucial role in the growth and metabolism of *W. anomalus* YWB-1. In this study, the highest production of β-damascenone was measured at an original Brix of 10%. In addition, a previous study showed that the content of β-damascenone produced by *C. saturnus* was positively related to the inoculum size (Methner et al., 2022). However, *W. anomalus* YWB-1 was observed to have a higher content of β-damascenone at moderate inoculum size (4.0%). This result indicated that the inoculum size was not an important factor influencing the synthesis of β-damascenone.

A culture medium volume test was performed to investigate the oxygen requirement of the strain to produce β-damascenone. A previous study showed that *W. anomalus* is an aerobic microorganism that prefers sufficient oxygen (Zha et al., 2013). In contrast, a lower oxygen content was more conducive to the production of β-damascenone by the yeast. However, when the culture medium volume was from 60 to 100 mL/250 mL, no significant difference in β-damascenone production was observed. Thus, the culture medium volume had a significant effect on *W. anomalus* replication rather than the production of β-damascenone. Moreover, compared with other fermentation parameters, the β-damascenone content produced by the yeast strain was lower under different culture medium volumes. Therefore, this factor was not considered for subsequent optimization experiments. In general, the highest concentration of 3.50 μg/L was obtained by OFAT, and the optimized conditions were an inoculum size of 4.0%, culture medium volume of 80 mL, initial pH 3.5, 10.0% original Brix, 35°C with shaking conditions, and 48-h incubation time.

### Screening of significant variables using a Plackett-Burman design

Plackett-Burman is used as a screening design to reduce experimental trials to *n*+1, where “*n*” is the number of variables in this study (Bhaturiwala et al., 2022). During the experimental runs, the observed β-damascenone contents ranged from 2.02 to 6.99 μg/L (Table 2). The highest production observed was 6.99 μg/L, which was increased by approximately two-fold compared with the OFAT method (3.50 μg/L).

**TABLE 2:** Plackett-Burman (PB) design experiments for screening the significant independent variables for the yield of β-damascenone from *Wickerhamomyces anomalus* YWB-1.

Run	A: initial pH	B: incubation temperature (°C)	C: inoculum size (%)	D: fermentation period (h)	E: original Brix (%)	β -damascenone (μg/L)
1	3.50	35	2	72	10	4.48 ± 0.30
2	6	35	4	24	5	3.66 ± 0.51
3	3.50	30	4	24	5	5.46 ± 0.12
4	3.50	30	2	72	5	4.19 ± 0.28
5	3.50	35	4	72	5	3.38 ± 0.81
6	3.50	35	4	24	10	6.99 ± 0.70
7	4.75	32.5	3	48	7.5	3.50 ± 0.33
8	4.75	32.5	3	48	7.5	3.26 ± 0.13
9	3.50	30	2	24	10	6.18 ± 0.80
10	6	35	4	72	10	2.47 ± 0.43
11	6	30	2	72	10	2.48 ± 0.13
12	6	35	2	24	10	3.84 ± 0.65
13	6	30	4	24	10	3.29 ± 0.66
14	4.75	32.5	3	48	7.5	3.70 ± 0.46
15	6	35	2	72	5	2.36 ± 0.02
16	3.50	30	4	72	10	5.10 ± 0.17
17	6	30	2	24	5	2.91 ± 0.75
18	3.50	35	2	24	5	4.45 ± 0.91
19	6	30	4	72	5	2.02 ± 0.46

The statistical significance of model terms was evaluated by their respective probability (*p*) value. The *p*-value of the model was 0.044 (Table 3), indicating that this model was significant. According to the *p*-values of the five factors evaluated, the significant factors affecting the yield of β-damascenone production were initial pH (A), fermentation period (D), and original biomass (E) at the 5% level of significance. The order of influence was A > D > E. However, other variables, such as B (incubation temperature) and C (inoculum size), were insignificant in the fermentation process. The performed model had good model fitness with a correlation coefficient of 0.9795. The adjusted *R*^2^ was 0.8769, representing a reasonable value. The production of β-damascenone may be best predicted by the following predictive modeling:


$$R=+3.88-1.08^{*}\,A+0.000^{*}\,B+0.092^{*}\,C-0.64^{*}\,D$$

$$+0.40^{*}\,E+0.20^{*}\,A^{*}\,B-0.11^{*}\,A^{*}\,C+0.097^{*}\,A^{*}\,D$$

$$-0.26^{*}\,A^{*}\,E+0.079^{*}\,B^{*}\,C-0.14^{*}\,B^{*}\,D+0.091^{*}\,B^{*}\,E$$

$$-0.16^{*}\,C^{*}\,D+0.016^{*}\,C^{*}\,E-0.077^{*}\,D^{*}\,E$$

**TABLE 3:** Analysis of variance (ANOVA) analysis of Plackett-Burman (PB) design experiment for β-damascenone production from *Wickerhamomyces anomalus* YWB-1.

Source	Coefficient	*F*-value	*p*-value	Significant
Model	3.88	42.51	0.044	*
A-initial pH	−1.08	380.98	0.0027	**
B-incubation temperature	0.000	0.000	1	
C-inoculum size	0.092	2.82	0.4842	
D-fermentation period	−0.64	36.38	0.0116	*
E-original Brix	0.40	8.13	0.0412	*
AB	0.20	8.68	0.1778	
AC	−0.11	4.24	0.4090	
AD	0.097	3.71	0.4631	
AE	−0.26	6.94	0.1124	
BC	0.079	0.37	0.5466	
BD	−0.14	1.37	0.3219	
BE	0.091	0.013	0.4896	
CD	−0.16	2.84	0.2623	
CE	0.016	1.31	0.8977	
DE	−0.077	3.01	0.5525	

*p < 0.05, **p < 0.01.

In the equation, *R* represents the total content of β-damascenone (μg/L), and A–E are the coded factors. Based on ANOVA and the calculated *t*-test, initial pH (*p* = 0.0027), fermentation period (*p* = 0.0116), and original Brix (*p* = 0.0412) were the most significant variables and were optimized using RSM to improve the yield of β-damascenone. The steepest ascent experiment was designed to approach the maximum response area of A, D, and E based on the PB design experiment results, as shown in Table 4. The β-damascenone production was the highest (6.48 μg/L) under the condition of Group 3, which was taken into consideration as the center point to implement the BBD experiment.

**TABLE 4:** Experiment design of steepest ascent and corresponding results.

Run	A: initial pH	D: fermentation period (h)	E: original Brix (%)	β -damascenone (μg/L)
1	2.5	0	6	1.57 ± 0.63
2	3	24	8	5.64 ± 0.51
3	3.5	48	10	6.48 ± 0.59
4	4	72	12	6.04 ± 0.32
5	4.5	96	14	4.20 ± 0.08

### Optimization of significant factors by Box-Behnken design

**FIGURE 4:**
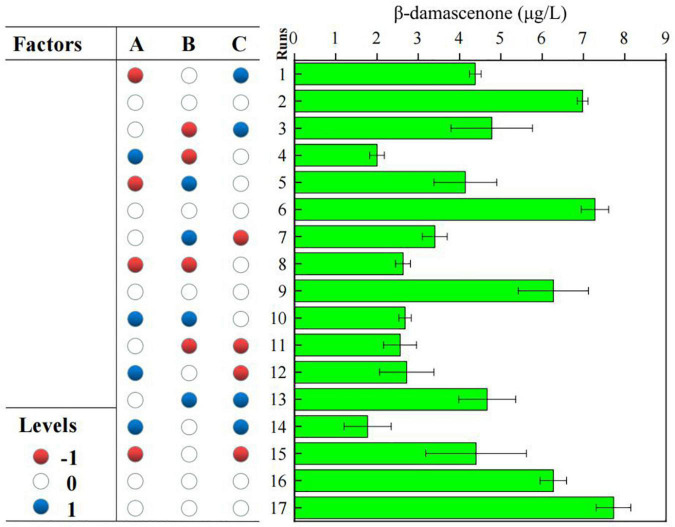
Experimental design based Box-Behnken design (BBD) model of response surface methodology (RSM) for the optimization of β-damascenone production.

To further optimize the screening result of the PB design, the BBD was employed to define the optimum levels of these three parameters for β-damascenone production. The three-factor [initial pH (A), original biomass (B), and fermentation period (C)] and three-level experimental design of 17 trials and results are presented in Figure 4. The maximum production of β-damascenone was observed around the center points of the experimental trials (6.27–7.73 μg/L), indicating that the optimum value of variables obtained by the steepest ascent experiment was within the tested high and low range of the test. The relationship between the response of the β-damascenone contents was represented by the second-order polynomial equation using multiple regression analysis. Independent variables were tested as follows:


$$R=+6.91-0.80^{*}\,A+0.36^{*}\,B+0.32^{*}\,C-0.21^{*}\,A^{*}\,B$$

-0.23*A*C-0.23*B*C-2.29*A-21.76*B-21.30*C2

Here, R represents β-damascenone contents, and A, B, and C were coded values for initial pH, original Brix, and fermentation period, respectively. Identification of model acceptability and statistically significant fermentation parameters were performed by ANOVA, as described in Table 5. The *p*-value for the response surface quadratic polynomial model of β-damascenone production was 0.0051, which indicated that the model was significant. Additionally, “the lack of fit” is a valid assessment test used to evaluate the reliability of a model, and a non-significant lack of fit is desirable (Yazici et al., 2021). In this model, the lack of fit (*p* = 0.1779) was not significant for the pure error, indicating that the model was fit. The linear effect of the initial pH was considered a significant factor in this model (*p* < 0.05) compared to the fermentation period and original Brix (*p* > 0.05), indicating that the initial pH had a significant effect on β-damascenone production. The correlation coefficient *R*^2^ was used to assess the accuracy of the established regression models developed, and the closer the value is to unity, the more accurate the response value estimated by the model (Qu et al., 2017). In this case, the correlation coefficient of *R*^2^ was 0.9157, indicating the accuracy of the regression model. The adjusted *R*^2^ was 0.8074. This result showed that the experiment was very reliable.

**TABLE 5:** Analysis of variance (ANOVA) for response surface quadratic model for the optimization of β-damascenone production.

Source	Sum of squares	*F*-value	*p*-value	Significant
				
			Prob > *F*	
Model	54.32	8.45	0.0051	**
A-initial pH	5.11	7.16	0.0317	*
B-original Brix	1.06	1.49	0.2623	
C-fermentation period	0.80	1.11	0.3261	
AB	0.17	0.24	0.6393	
AC	0.22	0.31	0.5968	
BC	0.22	0.31	0.5971	
A2	22.07	30.90	0.0009	**
B2	12.98	18.17	0.0037	**
C2	7.09	9.93	0.0161	*
Lack of fit	3.36	2.73	0.1779	not significant

*p < 0.05, **p < 0.01.

**FIGURE 5:**
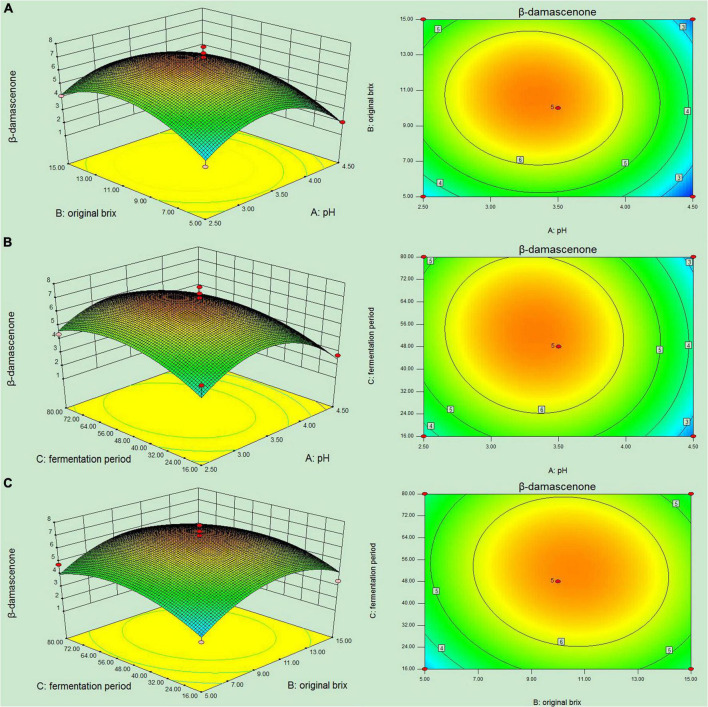
Response surface (3D) and contour plots (2D) for the β-damascenone content. **(A)** Effects of the initial pH and original Brix on the β-damascenone content. **(B)** Effects of the initial pH and fermentation period on the β-damascenone content. **(C)** Effects of the original Brix and fermentation period on the β-damascenone content.

The three-dimensional (3D) curve and contour plots were plotted to illustrate the interactive effect of the variables (Figure 5). In Figure 5A, the plotted graph of the fermentation period kept at the center point showed the interactive effect of the initial pH and original Brix. From the graphics, a higher β-damascenone yield was strongly supported when the initial pH and original Brix were maintained between 3.0 and 4.0 and 8.0 and 12.0%, respectively. In Figure 5B, the fermentation period was in the range from 40 to 64 h, which favored high β-damascenone production by the yeast strain. In addition, the interaction of the initial pH with the fermentation period favored the yield from +1 to −1 of the coding range. However, these two variables reduced the yield at their +1 range compared to the −1 range of variation. The circle on the plot representing the optimized values was located in the selected range of the two variables. The combined effects of fermentation period and original Brix were presented in Figure 5C. The interaction of these two variables showed that the optimum value of β-damascenone production ranged from 0 to +1 of the coded range.

Finally, the statistical model designed for β-damascenone production was validated. Using the point prediction function of Design Expert software, the predicted sets of optimized conditions that provided the maximum value of the β-damascenone concentration were obtained to test the model validity. The optimum conditions for β-damascenone production were predicted to be an initial pH of 3.31, an original Brix of 10.53%, and a fermentation period of 52.13 h with a maximal β-damascenone content of 7.30 μg/L. The experiment was performed under the optimum conditions to validate the accuracy of these prediction models, and the yield of β-damascenone was 7.25 ± 0.15 μg/L. The experimental result was very close to the predicted value, demonstrating the validity of the prediction model. Moreover, the highest yield of β-damascenone was greater than that of other non-*Saccharomyces* yeasts, such as *C. saturnus* (Methner et al., 2022), demonstrating that *W. anomalus* YWB-1 was a potential yeast for the production of β-damascenone. In addition, due to the complexity of microbial metabolism in the *Baijiu* fermentation process, in the industrial-scale *Baijiu* fermentation workshop, the microbial species and environmental factors are more complex, and more research will be needed to confirm this model.

## Conclusion

In this study, we revealed the changes in β-damascenone content during the light-flavor *Baijiu* brewing process and found that *W. anomalus* YWB-1 represents a potentially important yeast in the production of β-damascenone. Moreover, the initial pH, fermentation period, and original Brix exhibited significant effects on β-damascenone production. Additionally, the PB design and BBD of RSM were experimentally validated as suitable methods to optimize the production conditions of β-damascenone. Based on this method, we established a prediction model for the production of β-damascenone and significantly improved β-damascenone production compared to the OFAT approach, which was further validated by actual experiments. This work provides an efficient strategy for improving the contents of flavor compounds in *Baijiu* fermentation through the optimization of fermentation parameters.

## Data availability statement

The original contributions presented in the study are included in the article/Supplementary material, further inquiries can be directed to the corresponding author.

## Author contributions

JT, WJ, GZ, and QC participated in experimental processes. JT and BL wrote the manuscript. LZ and QL helped to modify the figures and tables. SC, QY, and SY provided the assistance and guidance throughout the research. SC assisted the manuscript checking. All authors contributed to the article and approved the submitted version.
