# Supplementary material for: Screening of β -damascenone-producing strains in light-flavor Baijiu and its production optimization via response surface methodology
Source: Front Microbiol. 2022 Nov 29;13:1067671. doi: 10.3389/fmicb.2022.1067671 (PMC9745179; doi:10.3389/fmicb.2022.1067671)
Supplement: Supplementary file 1 [file Data_Sheet_1.docx]

Screening of β-damascenone-producing strains in light‑flavor *Baijiu* and its production optimization via response surface methodology

Jie Tang, Bin Lin, Wei Jiang, Qun Li, Liping Zhu, Gang Zhang, Qianjin Chen, Qiang Yang, Shengzhi Yang and Shenxi Chen^*^

*Hubei Key Laboratory of Quality and Safety of Traditional Chinese Medicine* *& Health Food, Jing Brand Co., Ltd,* *Daye, China*

**Contents** **Page**

1. Figure S1. The related generation pathway of β-damascenone 3
2. Figure S2. Yeast strains obtained by a two-step method screening 4
3. Table S1. Parameters and variables used for β-damascenone content using OFAT

method 5

1. Table S2. Parameters and variables used for β-damascenone content using the 2-Level Factorial design 6
2. Table S3. Parameters and variables used for β-damascenone content using BBD 7


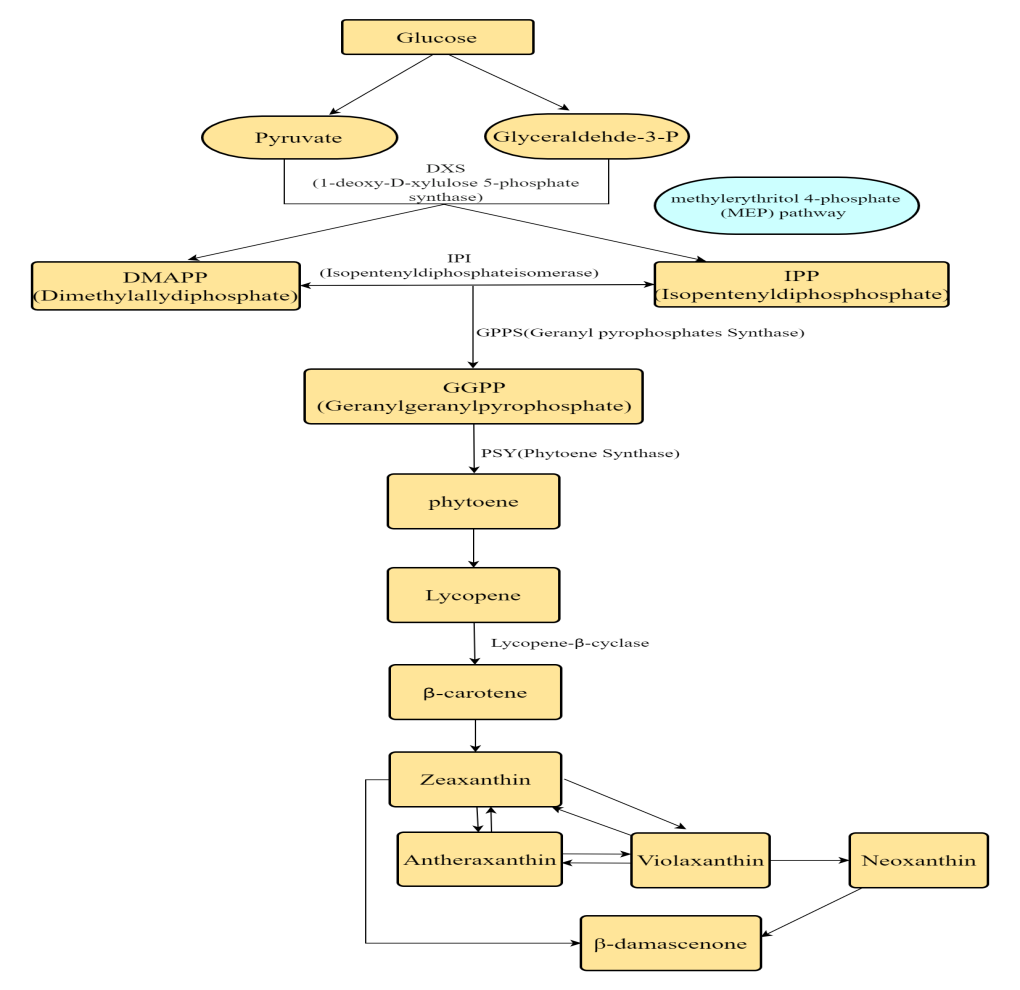


Figure S1. The related generation pathway of β-damascenone.


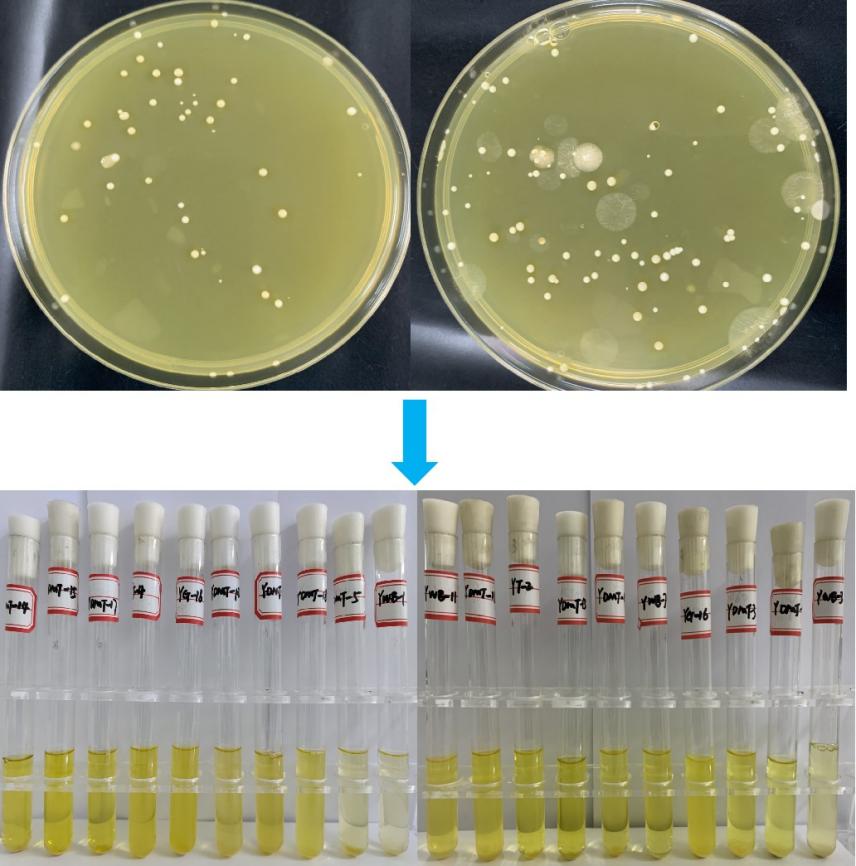


Figure S2. Yeast strains obtained by a two-step method screening

Table S1. Parameters and variables used for β-damascenone content using OFAT method.

| Variables | Conditions |
| --- | --- |
| Agitation condition | Static/shaking |
| Inoculum size % (v/v) | 2.0, 4.0, 6.0, 8.0, 10.0 |
| Initial pH  (before sterilization) | 3.0, 3.5, 4.0, 4.5, 5.0, 5.5, 6.0 |
| Original Brix (%) | 8.0, 10.0, 12.0, 14.0, 16.0, 18.0 |
| Incubation temperature (°C) | 20, 25, 30, 35, 40 |
| Medium volume (mL) | 20, 40, 60, 80, 100 |
| Incubation period (h) | 24, 48, 72, 96, 120 |

Table S2. Parameters and variables used for β-damascenone content using the 2-Level Factorial design.

| Factor level | A: initial pH | B: incubation temperature (℃) | C: inoculum size (%) | D: fermentation period (h) | E: original Brix (%) |
| --- | --- | --- | --- | --- | --- |
| -1 | 3.5 | 30 | 2 | 24 | 5 |
| 1 | 6 | 35 | 4 | 72 | 10 |

Table S3. Parameters and variables used for β-damascenone content using BBD.

| Variables | Symbol coded | Range and levels | | |
| --- | --- | --- | --- | --- |
|  |  | -1 | 0 | 1 |
| Initial pH | A | 2.5 | 3.5 | 4.5 |
| Original Brix (%) | B | 5 | 10 | 15 |
| Fermentation period (h) | C | 16 | 48 | 80 |
